# An Unusual Presentation of a Large Calcified Thyroid Nodule

**DOI:** 10.1155/2018/1795028

**Published:** 2018-11-07

**Authors:** Umesh Jayarajah, Pradeep Wijerathne, Oshan Basnayake, Sanjeewa Seneviratne

**Affiliations:** ^1^Professorial Surgical Unit, National Hospital of Sri Lanka, Colombo, Sri Lanka; ^2^Department of Surgery, Faculty of Medicine, University of Colombo, Sri Lanka

## Abstract

This case highlights the rare occurrence of a large benign calcified nodule of the left lobe of the thyroid gland with associated obstructive symptoms. Unusually, the calcification was confluent with similar calcified nodules in the subcutaneous tissue plane. The symptoms were alleviated following a total thyroidectomy.

## 1. Introduction

Cystic degeneration with haemorrhage and subsequent calcification may be seen in long standing multinodular goitres. However, large calcified thyroid nodules causing symptoms of obstruction with tracheal deviation are not commonly reported in literature [1]. We report a patient with a large calcified thyroid nodule with associated obstructive symptoms and tracheal deviation. Unusually, the calcified nodule was seen extending to the subcutaneous tissue plane between the strap muscles.

## 2. Case Presentation

**Figure 1:**
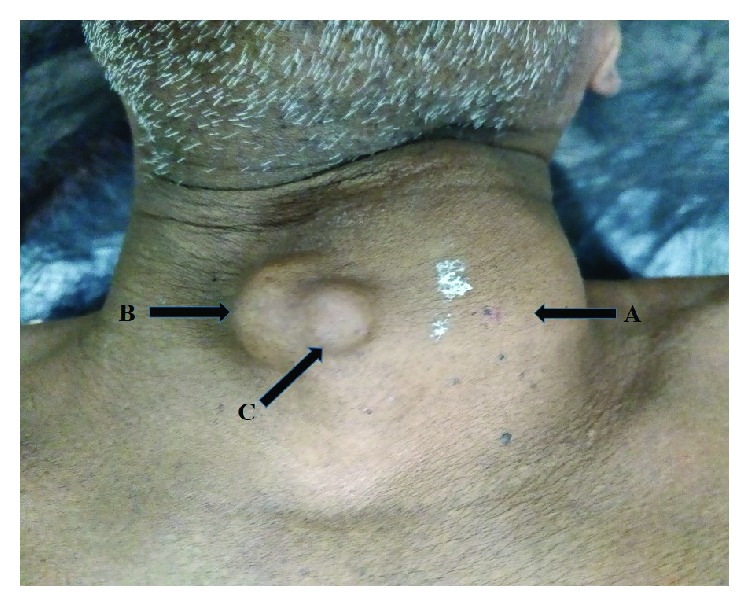
Figure showing the hard mass (arrow A) over the left hemithyroid measuring 8 cm (vertical) × 7 cm (transverse). Two tender, mobile lumps were palpable anterior to the mass in the subplatysmal (arrow B) and subcutaneous (arrow C) tissue planes.

**Figure 2:**
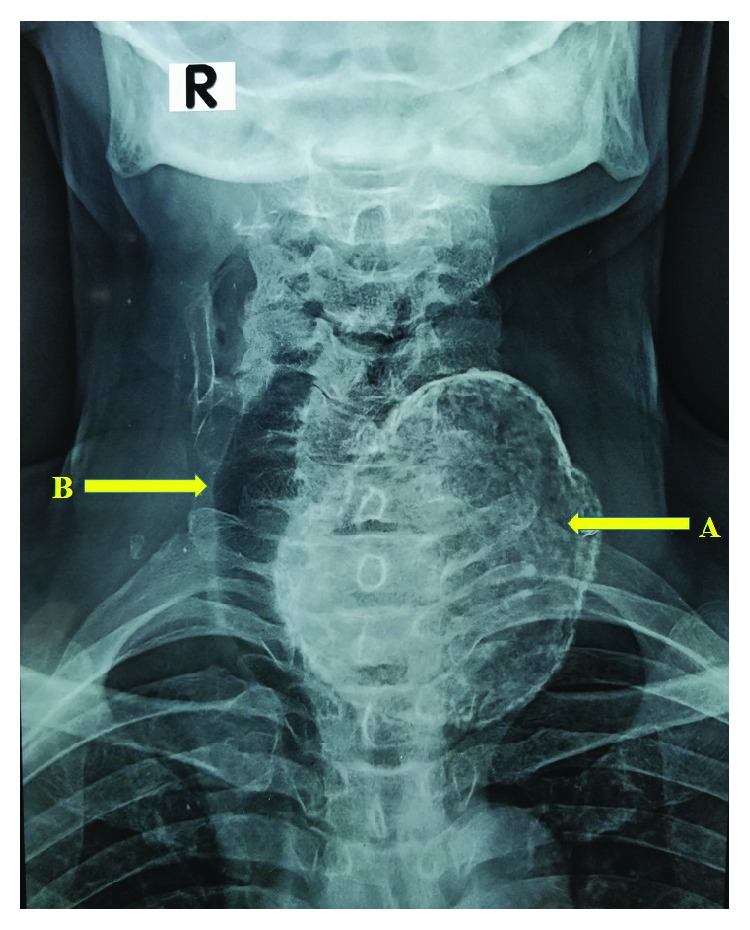
Neck X-ray radiography (anteroposterior view) showing the calcified left lobe (arrow A) with significant tracheal deviation to the opposite side (arrow B).

**Figure 3:**
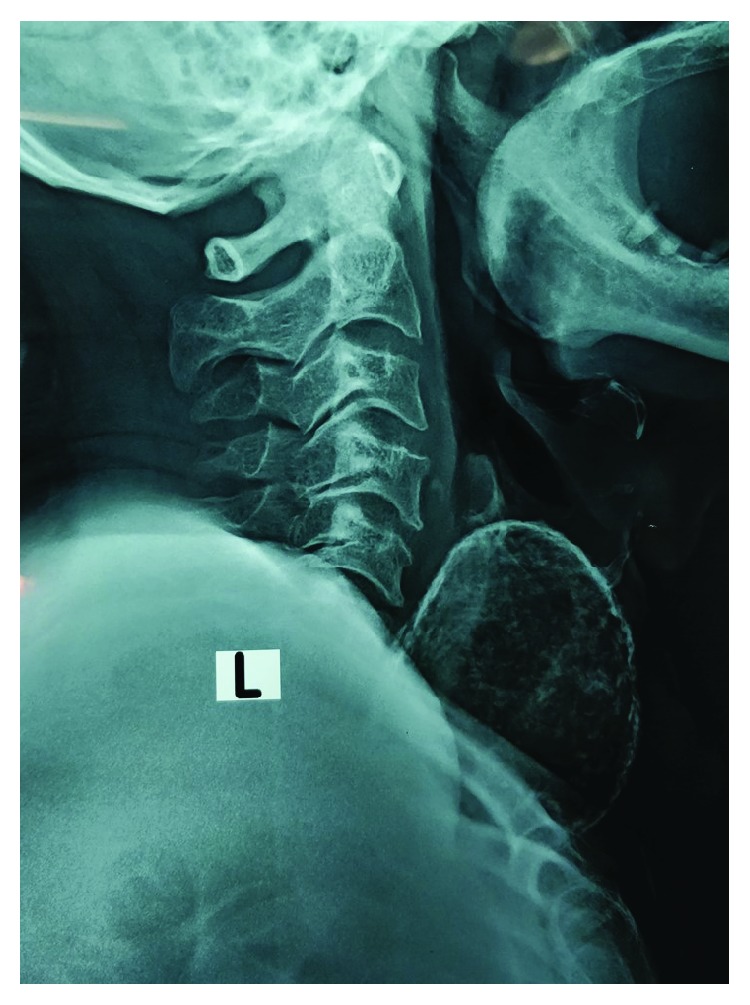
Neck X-ray radiography (lateral view) showing the calcified left lobe.

A 70-year-old Sri Lankan Tamil male with a history of well-controlled type 2 diabetes mellitus and a goitre of 30 years presented with a painful enlargement of goitre on the left side for one month. He had progressively worsening difficulty in breathing with intermittent dysphagia for solids. He did not have any symptoms of local infiltration and was clinically euthyroid. Examination revealed a hard mass arising from the left thyroid lobe measuring 8 cm × 7 cm in size with gross tracheal deviation to the right side. In addition, there were two mobile lumps anterior to the mass in the subcutaneous tissue plane (Figure 1). There was no retrosternal or retroclavicular extension on the left side. The right thyroid lobe was moderately enlarged and had multiple palpable nodules. There was no cervical lymphadenopathy. Ultrasound scan showed a large calcified left thyroid nodule and few superficial nodules. The outer surfaces of the nodules were delineated by an echogenic line suggestive of surface calcification. The internal echotexture of the nodules was not clearly appreciated due to artefacts from the surface calcifications. Right thyroid lobe showed only benign characteristics. Neck X-ray radiography showed a calcified left lobe with significant tracheal deviation to the opposite side (Figures 2 and 3). Thyroid stimulating hormone (TSH) and free thyroxine (T4) levels were within normal limits. Ultrasound-guided fine needle aspiration cytology (FNAC) showed scattered cyst macrophages, lymphocytes, and multinucleated giant cells in an eosinophilic background with scanty colloid. The features were compatible with a benign cyst (Thy 2).

**Figure 4:**
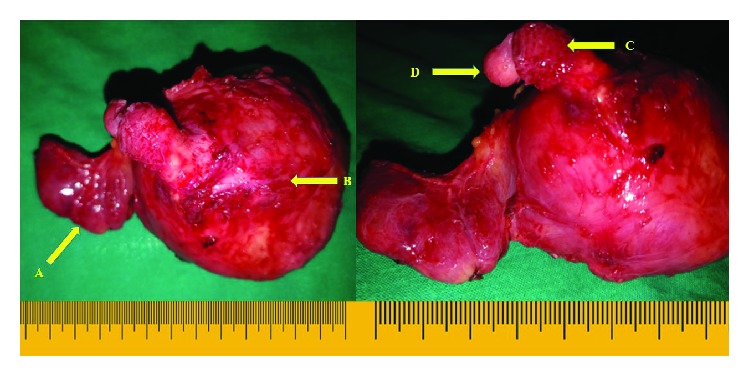
Total thyroidectomy specimen with the large hard calcified left lobe (arrow B) and nodular right lobe (arrow A) is shown. Two confluent nodules were seen in the subplatysmal (arrow C) and subcutaneous (arrow D) tissue planes extending through the deep fascia to the calcified left lobe.

The patient underwent a total thyroidectomy. Two confluent nodules were noted in the subcutaneous tissue plane extending through the deep fascia between the strap muscles to the calcified left lobe nodule. The deep fascia and strap muscles were thinned out and were adhered to the calcified left lobe (Figure 4). Division of strap muscles on the left side was required to mobilize and deliver the left lobe containing the calcified nodule.

Macroscopic assessment of the specimen consisted of the thyroid gland with the right lobe measuring 45 × 25 × 20 mm, the isthmus measuring 65 × 15 × 4 mm, and the enlarged left lobe measuring 80 × 75 × 55 mm. The outer surface of the gland was smooth. There were two confluent nodules over the anterior surface of the left lobe measuring 12 × 8 × 8 mm and 10 × 8 × 6 mm with a smooth outer surface.

Histology of the thyroid revealed an encapsulated left lobe lesion composed of a thick fibrous wall with foci of calcification. A dense inflammatory reaction comprising lymphocytes, foamy histiocytes, and scattered multinuclear giant cells was present within the capsule. The lumen was filled with amorphous, eosinophilic material with cholesterol clefts. A thin rim of compressed thyroid tissue was noted outside the fibrous wall. Sections from the confluent nodules revealed similar histopathological features and showed encapsulated lesion surrounded by a thin fibrous capsule. They were filled with numerous foreign body type giant cells and foamy histiocytes admixed with amorphous eosinophilic material and cholesterol clefts. No thyroid or lymphoid tissue was seen. The right lobe and isthmus showed features of a colloid storing goitre. There was no evidence of malignancy in the entire specimen. Overall, features of the main calcified nodule of the left lobe and the two smaller confluent nodules were compatible with a colloid cyst with secondary changes including calcification and chronic inflammation. The patient had an uneventful postoperative recovery with the alleviation of the obstructive symptoms. He was discharged on the first postoperative day on thyroxine 100 mg daily and remained healthy without any obstructive symptoms during a three-month routine outpatient clinic review.

## 3. Discussion

This case highlights the rare occurrence of a large calcified nodule of the thyroid gland which led to significant obstructive symptoms. Unlike other reported cases, this calcification was not associated with a malignancy [2, 3]. Furthermore, this had several features which differed substantially from commonly reported egg shell calcification. These include the size which is usually limited to a maximum diameter of 2 to 3 centimetres, focal distribution, and absence of compression symptoms, especially short duration rapidly progressive symptoms [4]. Our patient had a much larger single calcified nodule causing compression symptoms which progressed over a month period. Calcified nodules causing progressive symptoms over a short duration are rare as they tend to grow slowly over a long period. Furthermore, the pathological evaluation was negative for recent haemorrhage, acute inflammation, or malignancy which are the usual causes for rapidly progressive symptoms. Therefore, the reason for his progressive symptoms is not apparent. Also, in this patient, the calcification was confluent with similar calcified nodules in the superficial tissue planes which is an unusual phenomenon. The histological similarities with the main left lobe nodule suggest an origin from the thyroid gland.

Lyons et al. [1] described a similar patient with a large retrosternal calcified goitre which has had similar obstructive symptoms. Their patient had required a manubriotomy, due to the significant retrosternal extension during the total thyroidectomy [1]. However, the confluent calcified nodules in the superficial tissue plane are not previously reported in the literature. The reason for this unusual manifestation is not clear.

The majority of goitres with signs and symptoms of obstruction are benign, with approximately 50% being multinodular in origin [5]. Apart from the routine investigations, proper imaging with neck X-ray and CT or MRI may be utilised to determine the extent of the goitre. However, a decision was made to proceed without a CT scan as the nodule was mobile and there was no retrosternal extension.

Ultrasonography is important in characterising the goitre. If malignancy is suspected, a guided FNAC is indicated. In our patient, the FNAC was suggestive of a benign cyst. Thyroid cancers are increasingly being detected in both developing and developed countries [6], and in some situations, the diagnosis could be difficult based only on routine histology. Therefore, in doubtful cases, clinicians should also consider immunohistochemistry to decide on postsurgical management strategies, since both benign and malignant lesions can hide within calcified goitres [7].

## 4. Conclusion

Calcification of a large thyroid nodule is a rare phenomenon which may give rise to progressive obstructive symptoms. Unusually, in the patient reported here, the calcification was confluent with similar calcified nodules in the subplatysmal and subcutaneous tissue planes. The majority of such obstructive calcified goitres are benign. Imaging with chest and neck X-ray and CT or MRI may have to be utilised to determine the extent of the goitre before surgery.
